# The Functional Network of PrkC and Its Interaction Proteins in *Bacillus subtilis* Spores

**DOI:** 10.3390/microorganisms13040744

**Published:** 2025-03-26

**Authors:** Kangyi Mu, Tianlin Cui, Zequn Zhang, Yicong Shi, Chen Fang, Li Dong, Xiaosong Hu

**Affiliations:** 1 Key Laboratory of Fruits and Vegetables Processing, Ministry of Agriculture, Engineering Research Centre for Fruits and Vegetables Processing, Ministry of Education, National, Engineering Research Center for Fruit and Vegetable Processing, College of Food Science and Nutritional Engineering, China Agricultural University, Beijing 100083, China; mkys1999@163.com (K.M.); huxiaos@263.net (X.H.); 2 College of Food Science and Technology, Henan Agricultural University, Zhengzhou 450002, China

**Keywords:** *Bacillus subtilis*, spore germination, PrkC, interaction protein

## Abstract

In the food industry, food spoilage caused by spores is a pressing scientific challenge that needs to be addressed urgently, and spore germination is a key approach to solving this problem. Studies have shown that peptidoglycan-induced spore germination represents a novel mechanism of action, which can bind to the PASTA domain of the serine/threonine kinase PrkC. However, the signaling mechanism of peptidoglycan-induced spore germination remains unclear. This study focuses on *Bacillus subtilis*, using pull-down experiments to screen for proteins interacting with PrkC. There are 80 interaction proteins of PrkC that were identified in the spore. GO analysis reveals that PrkC-interacting proteins in the spore are mainly involved in metabolic processes, cell part and catalysis. KEGG results indicate that PrkC-interacting proteins in the spore are mainly involved in RNA degradation, quorum sensing, oxidative phosphorylation, etc. Additionally, proteins are categorized into six groups by function based on events that may be associated with post-germination triggered by peptidoglycan-induced activation of the PrkC signaling pathway, including “stimulate translation initiation” and “ATP synthesis and energy metabolism”. The experimental results provide a theoretical basis for further elucidating the signaling mechanism of PrkC, revealing the signaling pathway of peptidoglycan-induced spore germination, and identifying targeted inducers and repressors of spore germination.

## 1. Introduction

The spores of *Bacillus*, as the resting bodies, exhibit no metabolism and possess strong resistance to conditions and substances such as heat, radiation, dryness, and toxic chemicals. They can persist in the environment for extended periods, even years [1]. However, spores sense the environment and respond to favorable conditions by germinating and restoring cell growth [2]. Currently, research on spores primarily focuses on their germination mechanism, as germinated spores lose their resistance and become more susceptible to killing. Many *Bacillus* spores can cause food spoilage, food poisoning, and human diseases [3,4]. The dormant spores are non-pathogenic, and only synthesize toxins when they germinate and grow into vegetative cells [5]. Therefore, inhibiting spore germination or rapidly inducing germination of all spores holds significant research importance.

When the external environment becomes suitable, nutrients or non-nutrients can be utilized, and the spore exits dormancy to rapidly form a vegetative reproductive body [6]. In this process, spore germination is known as the key step. Spore germination is induced by a series of nutrient molecules, including amino acids, sugars, and cell wall peptides, which bind to receptors on the spore inner membrane and interact to activate downstream signaling substances, guiding spore resuscitation [7]. In the spore germination pathway, germination is induced mainly by nutrients and non-nutrients.

Previous studies have shown that spores possess a non-nutrient germination pathway through the PrkC protein kinase, which located on the inner membrane of *B. subtilis* spores can induce spore germination through binding to peptidoglycan (PGN) [8]. This germination pathway exists in the genera *Bacillus* and *Clostridium*, and is a new signal transmission pathway that is relatively independent of nutritional germination. Studies have shown that Δ*prkC* spores still responded to the nutrient germinant L-alanine and to the chemical germinant Ca^2+^-dipicolinic acid (Ca-DPA). Spores with low expression levels of germination receptors (GRs), activated by L-alanine and AGFK (a mixture of l-asparagine, d-glucose, d-fructose, and potassium ions), can germinate via the PrkC pathway [9]. This indicates that during the germination process of spores, the PrkC receptor germination pathway is independent and can work together with the traditional nutritional receptor germination pathway. Although superdormant spores cannot be induced to germinate by GRs, they can be induced to germinate by PrkC receptors [10]. The PrkC-mediated germination pathway only a single identified receptor, represents a promising and tractable target for controlling spore outgrowth in food industry applications. Therefore, the study of the mechanism of action of the PrkC germination pathway is of great significance, which can improve the mechanism of spore germination and provide a theoretical basis for more effective spore killing in food industry.

PGN is a crucial cell wall polymer for bacteria, consisting of N-acetylglucosamine (GlcNac) and N-acetylmuramic acid (MurNar) sugar chains linked by β-1,4 glycosidic bonds and connected by short peptides [11]. Depending on the third amino acid in its peptide chain, PGN is classified into Lys-type and meso-diaminopimelic acid (DAP)-type. The muropeptide fragment of PGN can bind to the PASTA (consisting of 3 β sheets and an α helix) [12] domain of the serine/threonine kinase PrkC, activating the intracellular kinase functional domain and inducing spore germination [8,13]. Notably, it is the DAP-type muropeptide, rather than the Lys-type muropeptide, that plays a role [8]. The recognition process of PrkC for DAP-type peptidoglycan fragments is semi-mediated by DAP. DAP-type peptidoglycan can interact with an arginine (Arg) residue located at position 500 on the PASTA domain of the C-terminal of PrkC. Furthermore, mutation of the arginine residue completely inhibits the binding of muropeptide to PrkC [13]. However, only a few studies have reported molecules downstream of PrkC-mediated spore germination. The elongation factor G (EF-G) in *B. subtilis* possesses ribosomal GTPase activity and can be phosphorylated by PrkC, thereby affecting the germination process of spores, and this modification is likely necessary for germination in response to PG. Elongation Factor Tu (Ef-Tu) also as a substrate of PrkC and phosphorylated by PrkC [14,15]. In *Bacillus anthracis* spores, the enolase (Eno) is phosphorylated by the conserved PrkC which decreases the catalytic activity of Eno, thereby controlling the overall spore germination process [16]. Another study showed that phosphoglycerate mutase (Pgm) is also regulated by PrkC phosphorylation, which is important for spore germination [17]. The specific details of the downstream signaling pathway mediated by PrkC are currently unclear. Therefore, this study will use pull-down experiments to screen interaction proteins of PrkC kinase in spores, and establish the interaction network of PrkC kinase. These results could provide reference for revealing the mechanism of PrkC protein in inducing the spore germination pathway.

## 2. Materials and Methods

### 2.1. Strain and Plasmid

The *Bacillus subtilis* 168 and PY79 strains are preserved in our laboratory. The *prkC* gene was amplified from *Bacillus subtilis* str. 168 genomic DNA using primers PrkC-F (5′-CAGCAAATGGGTCGCCTCGAGGTGCTAATCGGCAAGCGGATCAGC-3′) and PrkC-R (5′-GTGGTGGTGGTGGTGGCGGCCGCTTCATCTTTCGGATACTCAATGGT-3′). Both the purified PCR product and pGEX-4T-1 vector were digested with NcoI and XhoI. Ligate *prkC* gene and plasmid by Gibson assembly, and validated by Sanger sequencing.

*B. subtilis* 168 were cultured in LB broth medium until an optical density at 600 nm (OD_600_) of 1–2. Spores were grown for around 1 day on DSM medium and purified using the Histodenz gradient centrifugation method [18]. Spores were kept at −20 °C.

### 2.2. Induced Expression of PrkC Protein

The correctly sequenced plasmids were transferred into *E. coli* expression strain BL21, respectively. Positive monoclonal plaques were selected and inoculated into 5 mL of LB medium containing the corresponding antibiotics. The culture was incubated overnight at 37 °C and 220 rpm. The bacterial solution was inoculated into 200 mL of LB liquid medium containing the corresponding antibiotics at a volume ratio of 1:100, and incubated at 37 °C and 220 rpm until the OD_600_ reached 0.6–0.8. IPTG was added to the bacterial solution to a final concentration of 0.1 mM, and the culture was induced at 22 °C for 6–7 h. The bacterial solution was transferred to a new centrifuge tube, centrifuged for 10 min at 4 °C, and the supernatant was discarded. A buffer solution (100 mM Tris-HCl pH 7.5, 150 mM NaCl) was added to wash the bacterial cells, which were then centrifuged at 5000× *g* for 10 min at 4 °C, and the supernatant was discarded.

### 2.3. Purification of PrkC Protein

The precipitate was resuspended in 3 mL of lysis buffer (100 mM Tris-HCl pH 7.5, 150 mM NaCl, 1 mM PMSF). Following complete resuspension, ultrasonic disruption was performed at an output power of 270 W using 6–8 cycles of 20 s pulses interspersed with 40 s cooling intervals. The lysate was centrifuged at 12,000× *g* for 30 min at 4 °C, and the supernatant was transferred to a fresh centrifuge tube. For His-tagged protein purification, a Chelating Sepharose Fast Flow nickel affinity chromatography column was employed. Briefly, 2 mL of resin slurry was packed into a column and equilibrated with 10 mL of lysis buffer. The clarified supernatant was loaded onto the equilibrated column and incubated with the resin at 4 °C for 2 h. The flow-through was collected, and the column was sequentially washed with 10 column volumes of wash buffer (100 mM Tris-HCl pH 7.5, 150 mM NaCl, 50 mM imidazole) to remove nonspecifically bound impurities. Target protein elution was achieved using elution buffer (100 mM Tris-HCl pH 7.5, 150 mM NaCl, 300 mM imidazole), with five 2-mL fractions collected. Protein concentrations in the eluted fractions were quantified via Bradford assay (Coomassie Brilliant Blue G-250), and purification efficiency was assessed by SDS-PAGE analysis (12% resolving gel, Coomassie R-250 staining).

### 2.4. Pull-Down Experiment

Fusion-expressed GST empty-vector and GST-PRKC bacteria were obtained, treated with PDS solution, vortexed, and briefly centrifuged. After removal of the supernatant, the pellet was rinsed, resuspended in lysis buffer containing protease inhibitors, and lysed by ultrasonication. The lysate was centrifuged, and the supernatant was collected as the input control. Pre-equilibrated glutathione resin was incubated with different protein mixtures to assess interaction specificity. GST resin was incubated with spore lysate as a negative control for nonspecific binding, GST-PRKC resin was incubated with lysis buffer only to confirm that binding requires spore proteins, and GST-PRKC resin was incubated with spore lysate as the experimental group. After incubation at 4 °C, spores were lysed separately under similar conditions, and their lysate was collected. Following incubation, the resin was washed thoroughly to remove unbound proteins, and bound proteins were eluted using 20 mM freshly prepared reduced glutathione. The eluate was stored at −80 °C as the pull-down product. A portion was mixed with loading buffer, heated, and analyzed by Western blot.

### 2.5. Western Blot Analysis

The input and pull-down products were mixed with an appropriate volume of 4× loading buffer and incubated in a boiling water bath for 10 min. The mixture was centrifuged at 12,000× *g* for 3 min at 4 °C, and the supernatant was subjected to Western blot analysis. Electrophoresis was performed at a constant current of 16 mA per gel until the bromophenol blue tracking dye migrated to the bottom of the gel. Following electrophoresis, the gel was carefully removed, and a transfer sandwich was assembled for electroblotting. Protein transfer was conducted at a constant voltage of 100 V for 1 h in an ice-water-cooled electrotransfer tank. Upon completion of transfer, the membrane was retrieved and placed in separate antibody incubation chambers. Blocking was performed by incubating the membrane with 5% (*w*/*v*) skimmed milk powder or 2% (*w*/*v*) bovine serum albumin (BSA) in 1 × TBST for 1 h on a horizontal shaker. After discarding the blocking solution, the membrane was briefly rinsed with 1 × TBST and transferred to an antibody incubation tray. The GST-specific primary antibody (1:6000 dilution in 1 × TBST) was applied, and the membrane was incubated overnight at 4 °C. The primary antibody was removed, and the membrane was washed three times with 1 × TBST (10 min per wash). A horseradish peroxidase (HRP)-conjugated secondary antibody (1:8000 dilution in 1 × TBST) was then added, followed by incubation at room temperature for 2 h. After three additional TBST washes (10 min each), residual buffer was absorbed using filter paper. The membrane was exposed to X-ray film in a light-tight cassette for signal detection.

### 2.6. LC–MS/MS Analysis

The collected protein solution was digested with trypsin to generate peptide segments, which were then dried. Each dried peptide sample was re-dissolved in a sample solution containing 0.1% formic acid and 2% acetonitrile and subjected to LC–MS/MS analysis with a 100-min LC gradient for protein identification and label-free quantification (LFQ). Mass spectrometry (MS) data acquisition was performed using an Orbitrap Fusion Lumos Tribrid Mass Spectrometer (Thermo Fisher Scientific, San Jose, CA, USA) coupled to an Ultimate 3000 RSLCnano UHPLC system Peptide trapping was conducted on an Acclaim PepMap 100 nanoViper column (75 μm × 20 mm) at a 5 µL/min flow rate for 5 min, followed by separation on an Acclaim PepMap RSLC nanoViper column (75 μm × 500 mm, C-18, 2 μm, 100 Å, Thermo Fisher) at 300 nL/min and 40 °C. The mobile phases consisted of 0.1% formic acid in water (solvent A) and 0.1% formic acid in acetonitrile (solvent B). A multi-step linear gradient was applied as follows: 5% B (0–4 min), 5% B (5 min), 50% B (45 min), 90% B (50 min), 90% B (55 min), and re-equilibration to 5% B (65 min). A data-dependent acquisition (DDA) strategy was employed with 3 *s* cycles, utilizing specific MS scan parameters. Full MS scans were performed in the Q Exactive analyzer at 70,000 resolving power to precisely determine peptide mass-to-charge ratios (*m*/*z*), Scan range: 350–1800 *m*/*z*. MS2 analysis targeted precursor ions at 70,000 resolving power. Selected peptide ions were isolated via quadrupole (0.7 *m*/*z* window), fragmented using high-energy collision dissociation (HCD, 27% normalized collision energy). Additionally, a dynamic exclusion window of 30 s was applied to improve spectral acquisition.

### 2.7. MS Data Analysis and Peptide Identification

Acquired raw MS data were analyzed using the *Bacillus subtilis* UniProt database (strain-specific entries) with Sequest HT (Proteome Discoverer 2.5) and MaxQuant (v2.0.3). Full tryptic peptides were searched with up to two missed cleavages allowed, and fixed modifications included phosphorylation, ubiquitination, methylation, acetylation, and glycosylation. Data were filtered for precursor ions with a signal-to-noise (S/N) ratio of at least 1.5, and peptide spectrum matches (PSMs) were refined using a delta Cn threshold of 0.05.

To control the false discovery rate (FDR), q-values were calculated at the PSM level using Percolator and subsequently at the peptide level using the Qvality algorithm. For protein identification, explicit statistical criteria were applied: candidate proteins were validated using Sum PEP protein scores and accepted as significant if they met a strict FDR threshold of 0.01, with an alternative relaxed threshold of 0.05 provided in order not to filter proteins.

### 2.8. Bioinformatics Analysis

Identified proteins were annotated using DAVID (v6.8) with Gene Ontology (GO) terms (biological process, molecular function, cellular component, and only result FDR *p* < 0.05 and raw *p* values < 0.05). Proteins were functionally annotated and categorized using the *Subti*Wiki database. (https://subtiwiki.uni-goettingen.de/, accessed on 15 February 2025). Pathway enrichment analysis was subsequently conducted via the KEGG database (https://www.kegg.jp/, accessed 20 January 2025).

## 3. Results

### 3.1. In Vitro Expression and Purification of PrkC Protein

**Figure 1:**
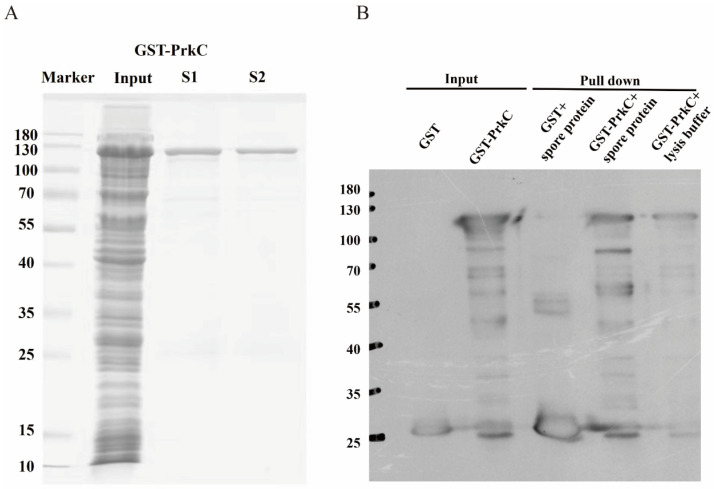
Purification of GST-PrkC and GST pull-down assay. (**A**) The SDS-PAGE of GST- PrkC. Lane Marker: Protein molecular weight marker; Lane 2: Input control; Lane 3 and 4: Elution once and twice. (**B**) Proteins were expressed in *E. coli* BL21(DE3) cells, and bacterial extracts were applied onto a glutathione resin. Empty plasmid GST+spore protein and GST-PrkC+ lysis buffer act as negative controls. Eluted proteins were probed with an anti-GST antibody by Western blotting.

A prokaryotic expression vector of GST-tagged PrkC protein was constructed, and GST-PrkC protein was purified (Figure 1A). The total proteins of *B. subtilis* spores were extracted, hybridized with purified GST-PrkC protein, and the proteins bound to PrkC protein were collected using GST antibody protein (Figure 1B). Then, the collected proteins were analyzed by liquid chromatography-mass spectrometry (LC–MS), and the data of these proteins were compared to analyze the signaling pathways and biological functions involved in them.

### 3.2. Screening of PrkC-Interacting Proteins in B. subtilis Spores

The LC–MS data were analyzed using ProteomeDiscoverer 2.4 against a target-decoy database of *B. subtilis* 168 (UniProt taxonomy ID 224308) with stringent filtering (1% FDR, ≥2 unique peptides). The data showed that in addition to PrkC protein, there were 79 interacting proteins in the spore (Table 1). Gene and protein names were annotated in the table.

### 3.3. GO Analysis of PrkC-Interacting Proteins in B. subtilis Spores

**Figure 2:**
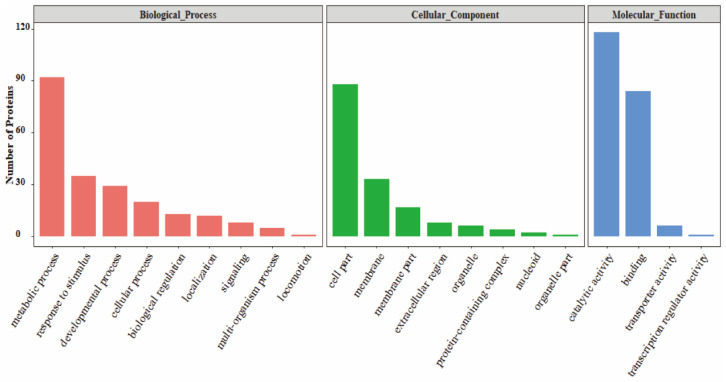
GO analysis of PrkC interaction proteins in *B. subtilis* spores.

To determine what biological characteristics these interacting proteins have, we performed GO analysis on the identified interacting proteins in the spores. The results showed (Figure 2) that the PrkC-interacting proteins in the spores were involved in numerous molecular pathways. The GO functional annotation mainly includes biological processes (BP), molecular functions (MF), and cellular components (CC). In biological processes, the interacting proteins in spore are involved in metabolic processes, stress responses, developmental processes, cellular processes, biological regulation, localization, signaling pathways, multi-tissue processes and movement. Among them, there are more PrkC-interacting proteins involved in the metabolic process in the spore, including energy metabolism, nucleotide metabolism, and biosynthesis (categorized via *Subiti*Wiki), which may be related to the restoration of physiological activity after germination. In cellular component, enriched in cell part, membrane and membrane part. PrkC localizes to the spore inner membrane and have a membrane-spanning sequence. Membrane proteins are more likely to interact with PrkC under dormant conditions. Molecular function analysis indicated that catalytic activity and binding described the majority of proteins in all four comparisons. The detailed functional classification analysis is systematically elaborated in the Discussion below.

### 3.4. KEGG Pathway Analysis and Protein–Protein Interaction Analysis of PrkC-Interacting Proteins in B. subtilis Spores

**Figure 3:**
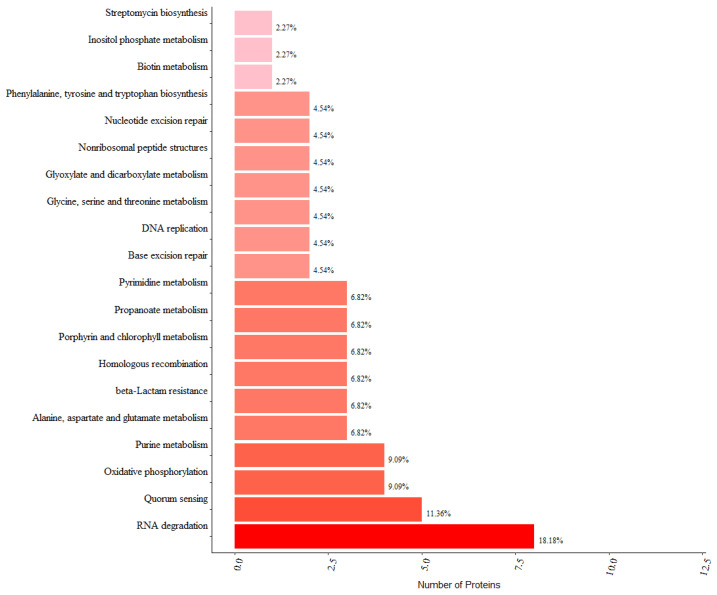
KEGG analysis of PrkC interaction proteins in *B. subtilis* spores.

**Figure 4:**
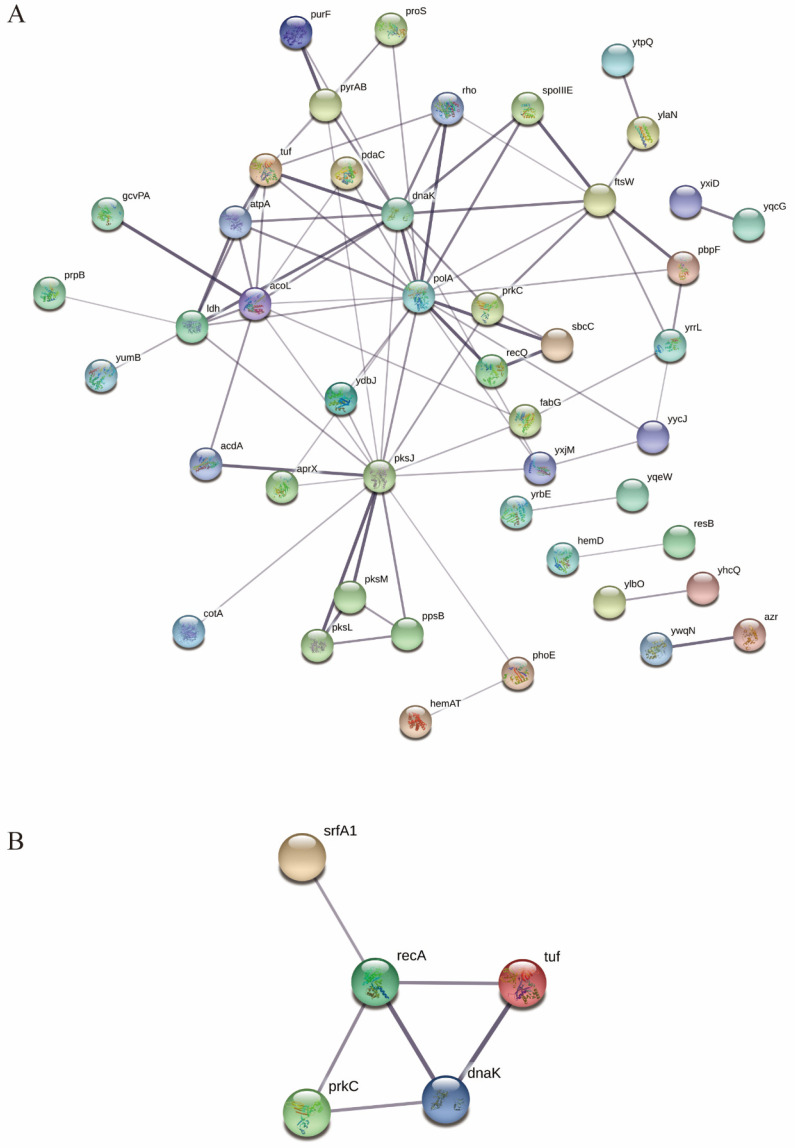
The role network diagram of PrkC and its interaction proteins in *B. subtilis* spores. (**A**) A protein–protein interaction (PPI) network was reconstructed using the STRING database. (**B**) Proteins unrelated to germination were pruned. The refined network revealed interaction between PrkC and two proteins.

In order to deeply analyze the mechanism of action of PrkC-interacting proteins, KEGG pathway analysis was carried out. The results showed (Figure 3) that in spores, interaction proteins of PrkC kinase are mainly involved in RNA degradation, population effect, oxidative phosphorylation, etc. More pathways are directed to metabolic processes such as amino acid metabolism and biotin metabolism. Consistent with GO’s analysis, suggesting that metabolism is important in this process. Association analysis of PrkC-interacting proteins in spores showed that *prkC* had direct interactions with *ftsW*, *sbcC*, *pksJ* and *dnaK*, respectively (Figure 4A). These interactions may occur more in vegetative cells or in the process of sporulation. Through optimizing the protein interaction network by removing non-central proteins and those functionally unrelated to dormant spore recovery activity, we identified *recA* and *dnaK* interacting with *prkC*. And these two also had some direct interactions, and both of them could interact with Tuf protein, while RecA protein could also interact with SrfA1 protein (Figure 4B). The functions of RecA and DnaK proteins have been extensively studied in *Bacillus subtilis*; however, their roles during spore germination remain poorly characterized. Both DNA repair and chaperone-mediated folding of newly synthesized proteins are important processes for restoring physiological activity following spore germination. These proteins may regulate the process of spore germination and thus deserve further investigation.

## 4. Discussion

Previous studies showed that PrkC mutant spores could not germinate under peptidoglycan-induced conditions, while GRs mutant spores could germinate, indicating that PrkC and GRs receptors were relatively independent in the spore germination pathway. In food sterilization, the two can interact with each other, which may produce a better sporicidal effect, and this result has been confirmed by the previous study of our group [19].Therefore study the downstream mechanism of PrkC-mediated germination is important for the guidance of food sterilization of spores. In this study, pull-down assay was used to screen the interaction protein of PrkC protein in the spores of *B. subtilis*.

The identification and mapping of protein interactions are critical for elucidation of their role in unraveling signaling pathways, metabolic networks, and regulatory cascades [20]. There are two main approaches for detecting interacting proteins, one measuring direct physical interactions and the second measuring interactions among groups of proteins. Co-immunoprecipitation (Co-IP) and pull-down assays are commonly employed to investigate proteomic interactions [21]. However, in the absence of specific protein antibodies, pull-down assays can serve as in vitro screening tools to initially identify unknown protein–protein interactions.

In spore germination, interaction proteins of PrkC kinase can be involved. Previous studies have shown that peptidoglycan can bind to the PASTA region of PrkC protein and activate downstream signaling molecules, so that the spore germination signal can be transmitted and promote spore germination. Functional annotation of the detected proteins reveals that the events that may be associated with post-germination triggered by peptidoglycan-induced activation of the PrkC signaling pathway can be categorized into the following categories. (i) “stimulate translation initiation”. Previous studies have found that the ribosomal GTPase EF-G is phosphorylated by PrkC, which stimulates translation of dormant spores and initiates spore germination. EF-G catalyzes the ribosome translocation of the mRNA–tRNA complex from the A and P sites to the P and E sites. Before that, aminoacyl-tRNA (aa-tRNA) is transported to the A site of the ribosome by the GTPase EF-Tu [22]. EF-Tu and EF-G exhibit sequential and essential functional coordination during ribosomal cycling. Moreover, in vitro PrkC specifically phosphorylates EF-Tu on T384 was previously reported [15]. The identification of the interaction of tuf (EF-Tu) with PrkC in our data suggests that PrkC-stimulated ribosomal activation is critical for spore germination. Several studies have found that dormant cells contain large amounts of mRNA and ribosomes [23,24]. Our data support that translation stimulation might be a fundamental mechanism of exit from dormancy [24]. (ii) “peptidoglycan metabolism”. Peptidoglycan cortex-degrading enzymes are typically released during the early phase of spore germination. Notably, we detected the presence of peptidoglycan synthesis-related proteins (e.g., PbpF, PdaC, and YrrL) [25,26] that may be activated by PrkC. A large PrkC-dependent effect of the *padC* gene has been demonstrated in vegetative cells [27]. We also detected a protein involved in the process of peptidoglycan peptide recovery, NagZ [28]. But no proteins associated with peptidoglycan degradation. Spore cortex-lytic enzymes essential for germination, such as SleB, CwlJ have been alternately demonstrated localized to the outer cortex (B) or the spore coat (J). They cannot directly interact with PrkC localized on the inner membrane. Thus, the signaling molecules for peptidoglycan degradation in the cortex after germination may not interact directly with PrkC. The mechanism inducing cortex degradation during the second stage of germination may be similar to nutrient-induced germination. (iii) “ATP synthesis and energy metabolism”. The NADH formation, oxygen uptake, and RNA synthesis were initiated immediately after germination [29]. We detected several proteins associated with energy metabolism (e.g., Ldh, AcoL, PrpB, YvoA, and Gdh) [30,31] and ATP synthase AtpA. PrkC has been shown to phosphorylate several metabolic enzymes in vitro [32], and initiation of metabolic processes with scarce resources in the spore is a prerequisite for spore germination. With the progress of germination, the spore requires substantial metabolic supplements. Multiple substrates may be involved in energy metabolism. Notably, Gdh is a glucose dehydrogenase that is directly involved in glucose metabolism and has the possibility of providing direct energy during the early metabolism of germination. Although studies have shown that neither of the two major glucose catabolic pathways is required for triggering *Bacillus subtilis* spore germination [30], Gdh may be important for energy provision. (iv) “nucleotide metabolism”. Accompanied by nucleotide metabolism in the early stages of germination [33]. We similarly detected a number of proteins involved in nucleotide metabolism (e.g., PurF and PyrAB). (v) “DNA repair”. Oxidative stress due to full hydration of the spore core during germination and activation of metabolism in spore outgrowth can lead to the production of reactive oxygen species (ROS) [34]. So DNA repair proteins in the spore may be extremely important in spore germination. We detected some DNA repair functional proteins including PolA, RecA, YprA (MrfA), YycJ, and SbcC. (vi) “ABC transporter”. During the early stages of spore germination, the involvement of transporter proteins is typically limited due to energy constraints. However, in the later stages of germination, numerous transporter proteins are recruited to facilitate the restoration of metabolic activity [33,35]. We detected ABC transporter AppD, YdbJ, and MntB. AppD, an oligopeptide transporter, may be activated by PrkC and potentially involved in protein transport processes during the late stages of spore germination.

The IM channel for Ca-DPA, composed of multiple SpoVA proteins (A, C, D, Eb, Ea, and F), opens to trigger a rapid release of Ca-DPA following spore germination [36]. Previous studies have suggested that SpoVAD acts as a similar cytoplasmic plug in the DPA transport complex [37]. During nutrient-induced germination, germinant receptor (GR) activation initiates cation efflux, which subsequently activates Ca-DPA channel opening [38]. SpoVAC acts as a mechanosensitive channel and allows the release of Ca-DPA and amino acids [39]. However, the mechanism by which muropeptide and PrkC binding leads to downstream Ca-DPA release remains unclear. We detected SpoVAD protein interacting with PrkC, though phosphorylation of SpoVAD has not been reported. Moreover, we detected YqhR (recently designated FigP), which oligomerizes with SpoVAF to form ion channels and serves as an essential cofactor for 5AF activity [40]. Protein phosphorylation, a critical signal transduction process, can induce conformational changes and plays a significant role in regulating spore germination [41,42]. Further studies are required to determine whether PrkC directly phosphorylates DPA channel components.

We also identified several spore-specific proteins, including SpoIIIE, YhcQ, CotI, and CotA. SpoIIIE is known to mediate chromosome translocation during sporulation [43], and its role in germination has not been reported. YhcQ, CotI and CotA are located on the spore coat. Notably, CotI is known as a bacterial spore kinase (BSK). They may be able to interact with specific substrates [44]. The tight association between some BSKs and glycosyl transferases and predicted nucleotide sugar metabolizing enzymes suggests that they may bind or phosphorylate one of these reactants [44]. Furthermore, as the earliest protein to contact with the signaling molecule, spore coat proteins could play a role in the inward transmission of the signaling molecule. However, this interaction should not occur on germination because of differences in localization in the spore. Additionally, we detected several proteins of particular interest in bacterial spores, such as the heat shock protein and molecular chaperone DnaK. Chaperones have been shown to participate in protein folding during early germination stages [45]. And DnaK is phosphorylated and dephosphorylated by PtkA and PtpZ in vegetative cells, respectively [46]. DnaK may also have a function in regulating protein quality in spore formation and germination.

The mechanism by which muropeptide binding to PrkC activates downstream germination events remains largely enigmatic, and our study offers critical clues to unravel this process.

## 5. Conclusions

In summary, this study successfully identified key proteins interacting with PrkC in *Bacillus subtilis* spores through pull-down assays, mass spectrometry, and comprehensive bioinformatics analyses. The findings revealed that these interacting proteins are involved in critical biological processes, such as metabolic process and are also enriched in many metabolic pathways. They were further categorized into six functional groups associated with post-germination events triggered by the peptidoglycan-induced PrkC signaling pathway, including “stimulate translation initiation”, “peptidoglycan metabolism”, “ATP synthesis and energy metabolism”, “nucleotide metabolism”, “DNA repair”, and “ABC transporter”. In addition, the discussion covers signal mechanisms that may induce Ca-DPA release and several proteins that, while crucial in vegetative cells, have been less extensively studied in the context of spore reactivation. These results enhance the understanding of the mechanisms underlying PrkC-mediated germination and provide a foundation for future investigations into specific signaling pathways.

## Figures and Tables

**Table 1:** PrkC interaction protein in *B. subtilis* spores.

UniprotKBc	Gene Names	Protein Names
P45867	*acdA*	Acyl-CoA dehydrogenase (EC 1.3.99.-)
O34324	*acoL*	Dihydrolipoyl dehydrogenase (EC 1.8.1.4) (Dihydrolipoamide dehydrogenase) (E3 component of acetoin cleaving system)
P42064	*appD*	Oligopeptide transport ATP-binding protein AppD
O31788	*aprX*	Serine protease AprX (EC 3.4.21.-)
P35146	*aroD*	3-dehydroquinate dehydratase (3-dehydroquinase) (EC 4.2.1.10) (Type I DHQase) (Type I dehydroquinase) (DHQ1)
P37808	*atpA*	ATP synthase subunit alpha (EC 7.1.2.2) (ATP synthase F1 sector subunit alpha) (F-ATPase subunit alpha) (Vegetative protein 100) (VEG100)
P07788	*cotA*	Laccase (EC 1.10.3.2) (Bilirubin oxidase) (EC 1.3.3.5) (Spore coat protein A)
O34656	*cotI*	Spore coat protein I
P17820	*dnaK*	Chaperone protein DnaK (HSP70) (Heat shock 70 kDa protein) (Heat shock protein 70)
P51831	*fabG*	3-oxoacyl-[acyl-carrier-protein] reductase FabG (EC 1.1.1.100) (3-ketoacyl-acyl carrier protein reductase) (Beta-Ketoacyl-acyl carrier protein reductase) (Beta-ketoacyl-ACP reductase)
O07639	*ftsW*	Probable peptidoglycan glycosyltransferase FtsW (PGT) (EC 2.4.99.28) (Cell division protein FtsW) (Cell wall polymerase) (Peptidoglycan polymerase) (PG polymerase)
P54376	*gcvPA*	Probable glycine dehydrogenase (decarboxylating) subunit 1 (EC 1.4.4.2) (Glycine cleavage system P-protein subunit 1) (Glycine decarboxylase subunit 1) (Glycine dehydrogenase (aminomethyl-transferring) subunit 1)
P12310	*gdh*	Glucose 1-dehydrogenase (EC 1.1.1.47)
O07621	*hemAT*	Heme-based aerotactic transducer HemAT
P21248	*hemD*	Uroporphyrinogen-III synthase (UROS) (EC 4.2.1.75) (Hydroxymethylbilane hydrolyase [cyclizing]) (Uroporphyrinogen-III cosynthase)
P13714	*ldh*	L-lactate dehydrogenase (L-LDH) (EC 1.1.1.27)
O34338	*mntB*	Manganese transport system ATP-binding protein MntB
O31667	*mtnX*	2-hydroxy-3-keto-5-methylthiopentenyl-1-phosphate phosphatase (HK-MTPenyl-1-P phosphatase) (EC 3.1.3.87)
P40406	*nagZ*	Beta-hexosaminidase (EC 3.2.1.52) (Beta-N-acetylhexosaminidase) (N-acetyl-beta-glucosaminidase) (N-acetylglucosaminidase) (ORF1)
P38050	*pbpF*	Penicillin-binding protein 1F (PBP-1F) (Penicillin-binding protein F) (Peptidoglycan TGase); Penicillin-sensitive transpeptidase (DD-transpeptidase)]
O34798	*pdaC*	Peptidoglycan-N-acetylmuramic acid deacetylase PdaC (Peptidoglycan MurNAc deacetylase) (EC 3.5.1.-) (Polysaccharide deacetylase PdaC)
O34819	*pelB*	Pectin lyase (PNL) (EC 4.2.2.10)
O07617	*phoE*	Uncharacterized phosphatase PhoE (EC 3.1.3.-)
P40806	*pksJ*	Polyketide synthase PksJ (PKS)
Q05470	*pksL*	Polyketide synthase PksL (PKS)
P40872	*pksM*	Polyketide synthase PksM
O34996	*polA*	DNA polymerase I (POL I) (EC 2.7.7.7)
P39846	*ppsB*	Plipastatin synthase subunit B (EC 2.3.1.-) (Peptide synthase 2) [Includes ATP-dependent tyrosine adenylase 1 (TyrA 1) (Tyrosine activase 1); ATP-dependent threonine adenylase (ThrA) (Threonine activase)]
O34507	*prkC*	Serine/threonine-protein kinase PrkC (Ser/Thr-protein kinase PrkC) (EC 2.7.11.1)
O31755	*proS*	Proline-tRNA ligase (EC 6.1.1.15) (Prolyl-tRNA synthetase) (ProRS)
P54528	*prpB*	2-methylisocitrate lyase (2-MIC) (MICL) (EC 4.1.3.-)
P00497	*purF*	Amidophosphoribosyltransferase (ATase) (EC 2.4.2.14) (Glutamine phosphoribosylpyrophosphate amidotransferase) (GPATase)
P25994	*pyrAB*	Carbamoyl phosphate synthase pyrimidine-specific large chain (EC 6.3.4.16) (EC 6.3.5.5) (Carbamoyl phosphate synthetase ammonia chain)
P16971	*recA*	Protein RecA (Recombinase A)
P17894	*recN*	DNA repair protein RecN (Recombination protein N)
P35161	*resB*	Cytochrome c biogenesis protein ResB
Q03222	*rho*	Transcription termination factor Rho (EC 3.6.4.-) (ATP-dependent helicase Rho)
O31466	*rtpA*	Tryptophan RNA-binding attenuator protein inhibitory protein (Anti-TRAP protein) (AT)
O06714	*sbcC*	Nuclease SbcCD subunit C
P21458	*spoIIIE*	DNA translocase SpoIIIE
P17867	*spoIVCA*	Putative DNA recombinase (Stage IV sporulation protein CA)
P40869	*spoVAD*	Stage V sporulation protein AD
P39627	*spsG*	Spore coat polysaccharide biosynthesis protein SpsG
P27206	*srfA1*	Surfactin synthase subunit 1
P40401	*ssuC*	Putative aliphatic sulfonates transport permease protein SsuC
O32165	*sufD*	Iron-sulfur cluster assembly protein SufD
P33166	*tuf*	Elongation factor Tu (EF-Tu) (P-40)
P54334	*xkdO*	Phage-like element PBSX protein XkdO
P37557	*yabO*	RQC P-site tRNA stabilizing factor (RqcP) (Hsp15) (Ribosome-associated protein quality control protein P)
O34772	*ycdC*	Uncharacterized protein YcdC
P96605	*ydbJ*	Uncharacterized ABC transporter ATP-binding protein YdbJ
O31557	*yfjB*	Uncharacterized protein YfjB
P54601	*yhcQ*	Spore coat protein F-like protein YhcQ
O07529	*yhdA*	FMN-dependent NADPH-azoreductase (EC 1.7.-.-) (Azobenzene reductase)
C0SP94	*yhfQ*	Putative ABC transporter substrate-binding lipoprotein YhfQ
O31607	*yjbI*	Group 2 truncated hemoglobin YjbI (Truncated Hb) (trHbO) (Hemoglobin-like protein YjbI) (Truncated BHb)
O31649	*yjdH*	Uncharacterized protein YjdH
O34798	*yjeA*	Peptidoglycan-N-acetylmuramic acid deacetylase PdaC (Peptidoglycan MurNAc deacetylase) (EC 3.5.1.-) (Polysaccharide deacetylase PdaC)
O07638	*ylaN*	UPF0358 protein YlaN
O34549	*ylbO*	Uncharacterized protein YlbO
O34569	*yoaA*	Uncharacterized N-acetyltransferase YoaA (EC 2.3.1.-)
O34748	*yocI*	ATP-dependent DNA helicase RecQ (DNA 3′-5′ helicase RecQ)
O31993	*yolB*	SPbeta prophage-derived uncharacterized protein YolB
P50830	*yprA*	Uncharacterized ATP-dependent helicase YprA (EC 3.6.4.-)
P45931	*yqbO*	Uncharacterized protein YqbO
P45942	*yqcG*	Toxin YqcG (DNase YqcG)
P54463	*yqeW*	Uncharacterized protein YqeW
P54516	*yqhR*	Uncharacterized protein YqhR
O05389	*yrbE*	Uncharacterized oxidoreductase YrbE (EC 1.-.-.-)
O34758	*yrrL*	Endolytic murein transglycosylase (EC 4.2.2.29) (Peptidoglycan lytic transglycosylase) (Peptidoglycan polymerization terminase)
O34496	*ytpQ*	UPF0354 protein YtpQ
C0SPA7	*yukB*	ESX secretion system protein YukB
O05267	*yumB*	NADH dehydrogenase-like protein YumB (EC 1.6.-.-)
O34817	*yvoA*	HTH-type transcriptional repressor NagR (N-acetylglucosamine utilization regulator)
P96726	*ywqN*	Putative NAD(P)H-dependent FMN-containing oxidoreductase YwqN (EC 1.-.-.-)
P42296	*yxiD*	Toxin YxiD (DNase YxiD)
P42299	*yxiG*	Uncharacterized protein YxiG
P55183	*yxjM*	Sensor histidine kinase YxjM (EC 2.7.13.3)
Q07835	*yxxF*	Uncharacterized transporter YxxF
C0SP91	*yycJ*	Exodeoxyribonuclease YycJ (EC 3.1.11.-)
P45867	*acdA*	Acyl-CoA dehydrogenase (EC 1.3.99.-)
O34324	*acoL*	Dihydrolipoyl dehydrogenase (EC 1.8.1.4) (Dihydrolipoamide dehydrogenase) (E3 component of acetoin cleaving system)
P42064	*appD*	Oligopeptide transport ATP-binding protein AppD

## Data Availability

The original contributions presented in this study are included in this article. Further inquiries can be directed to the corresponding author.
